# Perioperative use of specific coagulation factors in burn patients. an international survey

**DOI:** 10.1186/2197-425X-3-S1-A854

**Published:** 2015-10-01

**Authors:** A Lavrentieva, N Depetris, E Kaimakamis, M Stella, M Berardino

**Affiliations:** Papanikolaou Hospital, Thessaloniki, Greece; Burn Center, Turin, Italy

## Introduction

Burn injury is traditionally referred to as a common triggering cause for acute coagulopathy. Even though there is extensive literature exploring coagulopathy in trauma patients, few studies have focused on management of coagulopathy in specialized burn units.

## Objectives

The aim of this study was to evaluate physician views, considerations and practices regarding the perioperative use of specific coagulation factors in burn patients.

## Methods

Three hundred and fifty questionnaires were distributed via electronic mail to burn specialists. Participation in the survey was voluntary and anonymous. The questionnaire consisted of two parts: the first collected physician and institutional demographics, and the second explored the opinions and attitudes of the specialists regarding perioperative coagulopathy diagnosis and treatment.

## Results

There was a 16 % response rate. The majority of the responders (74.5%) stated that no specific scoring system is used in their department to detect coagulopathy. More than half (56%) of specialists from Europe and 82% of specialists from North America recommend treatment with fibrinogen concentrate or cryoprecipitate if significant bleeding is accompanied by thromboelastometric signs of a functional fibrinogen deficit or a plasma fibrinogen level of less than 1.5 to 2.0 g/L, while 64% of doctors from other regions do not use fibrinogen concentrate or cryoprecipitate. Prothrombin complex concentrate is the most commonly used specific coagulation factor in Europe (40%), but it is not commonly used in N. America (9%) and in other regions (7.1%). Thrombin and fibrin sealant are the most common topical factors applied intraoperatively in burn patients to stop bleeding in N. America (72.7% and 82%, respectively). The most common topical factor used intraoperatively in Europe is fibrin sealant (40%), while in other regions topical factors are rarely used (14.3 %).

## Conclusions

Burn ICU physicians do not use specific scoring system to detect coagulopathy in burn patients. Fibrinogen concentrate, prothrombin complex and cryoprecipitate are the most commonly used coagulation factors in bleeding burn patients in Europe and North America. Thrombin and fibrin sealant are the most common topical factors applied intraoperatively in burn patients to stop bleeding. Extremely low rate of specialists from other regions implements specific treatment options in burn patients with perioperative coagulopathy and bleeding.

